# The Effectiveness of Intensity Modulated Radiation Therapy versus Three-Dimensional Radiation Therapy in Prostate Cancer: A Meta-Analysis of the Literatures

**DOI:** 10.1371/journal.pone.0154499

**Published:** 2016-05-12

**Authors:** Ting Yu, Qiongwen Zhang, Tianying Zheng, Huashan Shi, Yang Liu, Shijian Feng, Meiqin Hao, Lei Ye, Xueqian Wu, Cheng Yang

**Affiliations:** 1 State Key Laboratory of Biotherapy and Cancer Center, West China Hospital, West China Medical School, Sichuan University, Chengdu, Sichuan, PRC; 2 State Key Laboratory of Biotherapy and Department of Head and Neck Oncology, West China Hospital, West China Medical School, Sichuan University, Chengdu, Sichuan, PRC; Innsbruck Medical University, AUSTRIA

## Abstract

**Background and Purpose:**

Intensity modulated radiation therapy (IMRT) can deliver higher doses with less damage of healthy tissues compared with three-dimensional radiation therapy (3DCRT). However, for the scenarios with better clinical outcomes for IMRT than 3DCRT in prostate cancer, the results remain ambiguous. We performed a meta-analysis to assess whether IMRT can provide better clinical outcomes in comparison with 3DCRT in patients diagnosed with prostate cancer.

**Materials and Methods:**

We conducted a meta-analysis of 23 studies (n = 9556) comparing the clinical outcomes, including gastrointestinal (GI) toxicity, genitourinary (GU) toxicity, biochemical controland overall survival (OS).

**Results:**

IMRT was significantly associated with decreased 2–4 grade acute GI toxicity [risk ratio (RR) = 0.59 (95% confidence interval (CI), 0.44, 0.78)], late GI toxicity [RR = 0.54, 95%CI (0.38, 0.78)], late rectal bleeding [RR = 0.48, 95%CI (0.27, 0.85)], and achieved better biochemical control[RR = 1.17, 95%CI (1.08, 1.27)] in comparison with 3DCRT. IMRT and 3DCRT remain the same in regard of grade 2–4 acute rectal toxicity [RR = 1.03, 95%CI (0.45, 2.36)], late GU toxicity [RR = 1.03, 95%CI (0.82, 1.30)] and overall survival [RR = 1.07, 95%CI (0.96, 1.19)], while IMRT slightly increased the morbidity of grade 2–4 acute GU toxicity [RR = 1.08, 95%CI (1.00, 1.17)].

**Conclusions:**

Although some bias cannot be ignored, IMRT appears to be a better choice for the treatment of prostate cancer when compared with 3DCRT.

## Introduction

Prostate cancer ranks the most common cancer and the second most common cause of cancer death in men [1]. Radiation therapy (RT) is widely used in the treatment of prostate cancer [2–6]. Dose escalation has been generally adopted in the RT of prostate cancer for its advantage of improved tumor control outcomes [7–14]. Since most of the patients who were diagnosed with non-metastatic prostate cancer can survive longer than 10 years, the choice of RT techniques with minimized RT-related toxicity is important for improving quality of life[15–19]. However, higher doses are linked to increased normal tissue toxicity, such as late gastrointestinal (GI) toxicity and late genitourinary (GU) toxicity [7,20].

As technology advances, new RT techniques have emerged and have been used in clinical practice. Three-dimensional conformal radiation therapy (3DCRT) delivers a radiation dose conforming to the target volume of tumor [21]. Thus 3DCRT significantly increases the target dose whilereducing the exposure of healthy tissue [2,21,22]. RT techniques evolved to an advanced form of 3DCRT, intensity modulated radiation therapy (IMRT), which generates non-uniform fields to increase the radiation dose delivered to the intended target volume while potentially minimizing the irradiation to the organs at risk [23,24]. Nevertheless, the probability of a marginal miss is a potential weakness of IMRT. Besides, the dosehomogeneity, increase of irradiation doses to larger volumes of healthy tissuesand longer time required for planning need to be considered in the application of IMRT[25,26]. The increased total body exposure and monitor units raise the risk of second malignancies of IMRT in comparison with conventional RT [27–30].

However, the potential benefits of IMRT over 3DCRT for prostate cancer treatment have not yet been clarified. Therefore, this meta-analysis was conducted to assess whether IMRT could improve clinical outcomes in comparison with 3DCRT in patients diagnosed with prostate cancer, including acute GI toxicity, acute GU toxicity, acute rectal toxicity, late GI toxicity, late GU toxicity, late rectal bleeding, biochemical controland overall survival (OS).

## Materials and Methods

### Primary search strategy

The PubMed (MEDLINE) and EMBASE were searched for relevant publications by combining search terms “prostate cancer [Title/ Abstract]”, “Intensity modulated radiation therapy [Title/ Abstract]”, “IMRT[Title/ Abstract]”, “Three dimensional conformal radiation therapy [Title/ Abstract]”, and “3DCRT [Title/ Abstract]”. There was no date of publication limits and the most recentliterature was published on July 25^th^, 2015. Only studies in English were included. Furthermore, reference lists from primarily identified studies were also manually searched.

### Criteria for considering studies in this review

Eligible studies had to compare IMRT with 3DCRT in patients diagnosed with prostate cancer. Those studies were then selected according to the following criteria: (1) Studies with GI, GU toxicity or other clinical outcomes, including RFS or OS, were included in this meta-analysis. (2) Late GI and late GU toxicity were scored according to the Fox Chase (FC) modification of the Radiation Therapy Oncology Group (RTOG) and Late Effects Normal Tissue Task Force (LENT) toxicity criteria (RTOG/FC-LENT late toxicity criteria)/Common Terminology Criteria (CTC) (version 2.0, 3.0 or 4.0) [31]. (3) Late rectal bleeding was scored based on RTOG criteria [32]. (4) Biochemical failure was defined as a rise in prostate-specific antigen (PSA) level of ≥ 2 ng/ml above the nadir, with no backdating (ASTROPhoenix definition) [33]. Two reviewers conducted a primary assessment independently to confirm the eligibility of the abstracts searched from database. Discrepancies were solved by cooperative discussion. The names of all authors and medical centers involved in each study were carefully examined in order to avoid duplicated data. If duplicated studies were found, the studies with the largest number of patients were retained.

### Data Extraction

Data were carefully extracted independently from all the included publications by two reviewers, using a standardized data collection form. Data extraction included the following items: author, year, study design, sample size, planning target volume (PTV), total dose of RT, fraction dose, margin, method for dose prescription, image guidance, tumor stage, median follow-up time, percentage of androgen deprivation therapy (ADT), andscore criteria.

### Statistical Analysis

Included publications were divided into eight groups for analysis: those with data regarding acute GI toxicity, acute GU toxicity, acute rectal toxicity, late GI toxicity, late GU toxicity, late rectal bleeding, biochemical control, and OS.

For the quantitative aggregation of outcomes, the impacts of treatment on acute toxicity, late toxicity, RFS and OS of each publication were reported for by estimating RRs with 95% confidence interval (CI) value. The RR and its 95% CI were extracted from the original article. If RR and its 95% CI were not available, the total number of events and number of patients at risk in each group were extracted to estimate RR and its 95% CI, according to the methods described by Parmer et al [34]. Eventually, Kaplan-Meier curves were read using Engauge Digitizer version 4.1 (free software downloaded from http://sourceforge.net) to extract data to reconstruct RR and its 95% CIwhen the exploitable data were only presented in the form of figure.

To assess the heterogeneity of the publications, a fixed effect model was used for meta-analysis. If the *I*^*2*^ was higher than 50%, a random effect model was used. Conventionally, the difference would be considered statistically significant if the 95% CI of RR did not overlap the value 1 with p < 0.05. Study estimates, together with pooled estimates, were presented in the form of forest plots. Publication bias was assessed graphically by funnel plots and Egger’s linear regression method was used to assess the funnel plot asymmetry. (p < 0.05 was considered to be statistically significant) [35]. The meta-analysis was done with Stata version 12.0 (Stata Corporation, College Station, TX, USA).

## Results

### Study selection and characteristics

**Fig 1:**
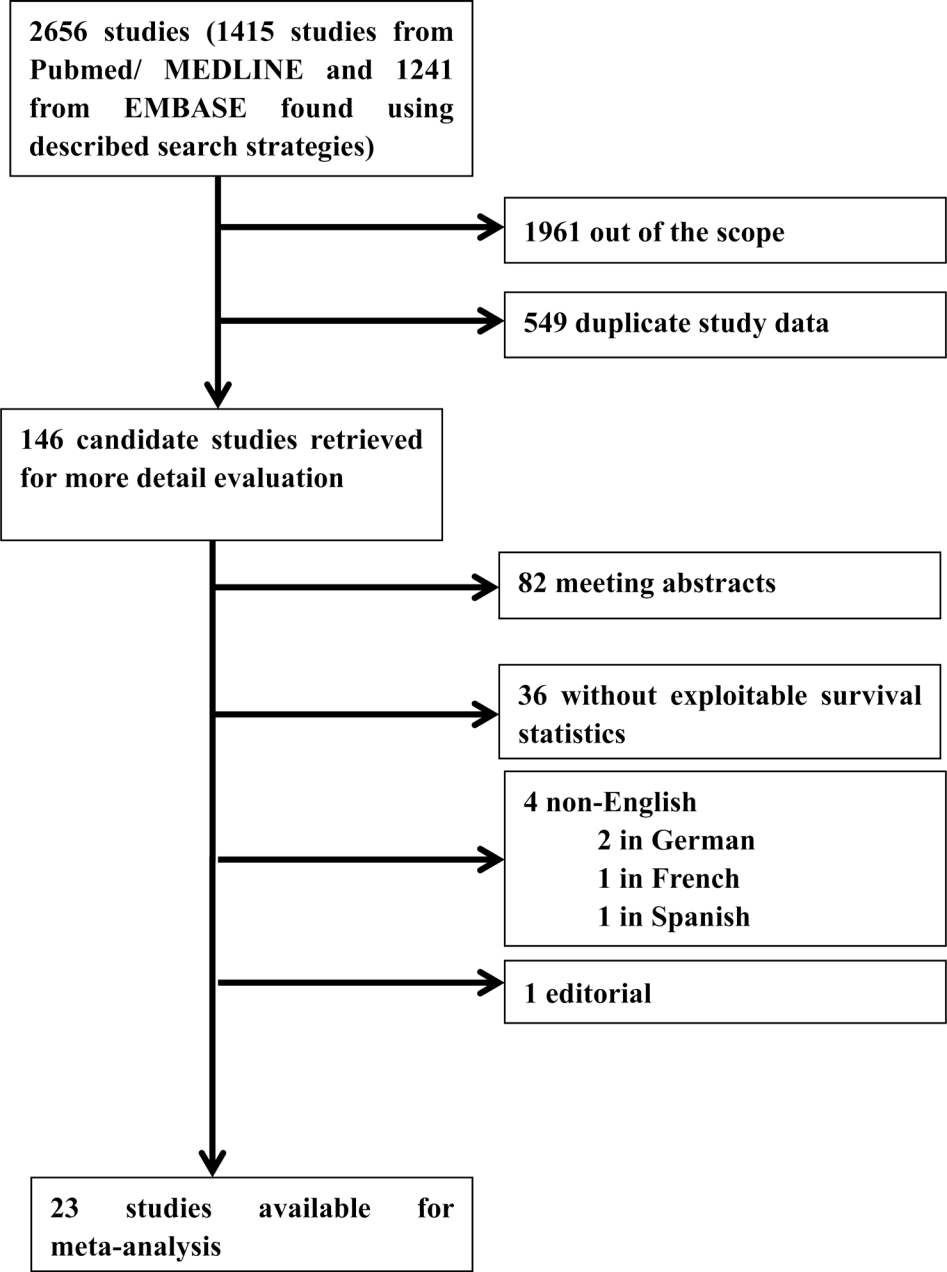
Flow chart of the literature search and selection of included studies.

The initial search algorithm retrieved 2656 references and 146 candidate studies were fully evaluated. Upon further review, 23 articles met the eligibility criteria, and the other 123 articles were out of scope. The flowchart of the literature search is shown in Fig 1.

The total number of the included patients was 9556, ranging from 27 to 1571 per study. The main characteristics of included studies are presented in Table 1. The study design was more often a retrospective (n = 16) than a prospective cohort study (n = 5). The prescribed doses to the primary tumor were 70–85.3 Gy in IMRT group and 55.8–84.8 Gy in 3DCRT group. Stage I/II comprised 77.3% of the patients, and the remaining 22.7% were in stage III/IV. The median follow-up time ranged from 5.3 months to 120 months.

**Table 1:** Summary of the studies included in the meta-analysis.

Author	Year	Study design	Number (3DCRT/IMTR)	PTV	total dose/fraction dose (Gy) (3DCRT VS IMRT)	Margin (mm)	Method for dose prescript-ion	Image guidance	ADT%(3DCRT/IMRT) & p value	Tumor stage I/II (III/IV)	Median follow-up (m)(3DCRT/IMRT)	score criteria
**post-operative RT (n = 2)**												
Alongi F[36]	2009	Retro.	172(81/91)	Prostatic bed, Pelvic nodes	72.1/1.8 **VS** 72.5/1.8	8	Isodose level	NO	61/56n.s.	NR/NR	3/3	RTOG toxicity scale
Goenka A[37]	2011	Retro.	285 (109/176)	NR	66-72/NR VS 66-72/NR	NR	NR	NO	100/100n.s.	NR/NR	97/53	RTOG toxicity scale,CTCAE version 3.0
**Primary RT (n = 21)**												
Ashman JB[38]	2005	Retro.	27 (14/13)	Prostatic bed, Pelvic nodes, seminal vesicles	75.6/1.8 **VS**81/1.8	10	Isocenter	NO	100/100n.s.	12/15	30/30	RTOG toxicity scale
Cho JH[39]	2008	Retro.	50 (35/15)	Prostatic bed	70.2/1.8 **VS**70/2.5	NR	Isocenter	NO	44/44n.s.	26/24	3/3	RTOG toxicity scale
Dolezel M[40]*	2010	Pro.	232 (94/138)	Prostatic bed, Pelvic nodes, seminal vesicles	74/2 **VS**78/2	10	Isocenter	NO	94.7/55	76/156	68.4/37.2	RTOG toxicity scale
Dolezel M[41]*	2015	Pro.	533 (320/233)	Prostatic bed, seminal vesicles	70-74/2 **VS**78-82/2	10	Isocenter	NO	40.3/62.3	332/221	104/60	RTOG toxicity scale, ASTROPhoenix definition
Forsythe K [42]	2011	Retro.	812 (521/291)	Prostatic bed,seminal vesicles	NR	10–12	Isocenter	Partly	87.9/75.9p <0.01	NR/NR	74.4/33.6	RTOG toxicity scale
Jani AB[43]	2007	Pro.	481(373/108)	Prostatic bed,seminal vesicles	68.5/1.8–2 **VS**75/1.8–2	10	NR	NO	53/51	413/68	NR/NR	RTOG toxicity scale
Kim H[44]	2014	Retro.	86 (56/30)	Prostatic bed, Pelvic nodes,seminal vesicles	70/1.8 **VS**70/2.5	5	Isocenter	NO	56.7/53.6n.s.	43/43	78.6/73.4	RTOG toxicity scale
Kupelian PA[45]*	2002	Retro.	282 (116/166)	Prostatic bed, Pelvic nodes, seminal vesicles	78/2 **VS**70/2.5	8–15	Isodose level	NO	72/60p = 0.049	263/19	25/25	RTOG toxicity scale, ASTROPhoenix definition
Odrazka K[46]*	2010	Retro.	340(228/112)	Prostatic bed, seminal vesicles	70/2 **VS**78/2	10–15	Isocenter	NO	19.7/54.5	NR/NR	70.8/36	RTOG toxicity scale
Ratnayake G[47]	2013	Pro.	103 (52/51)	Prostatic bed, Pelvic nodes, seminal vesicles	74 or 78/2 **VS**78/2	7–10	Isodose level	YES	31/59p = 0.06	83/19	48/38	RTOG toxicity scale
Sharma NK[48]	2007	Retro.	293(170/123)	Prostatic bed, Pelvic nodes, seminal vesicles	76/2 **VS**76/1.8	10 **VS** 3–5	Isodose level	NO	100/100n.s	223/70	86/40	RTOG toxicity scale
Someya M[49]	2015	Retro.	129 (55/74)	Prostatic bed, seminal vesicles	70/2 **VS** 78/2	10 **VS** 8	Isocenter	NO	83.6/70.3	104/25	85/38	RTOG toxicity scale
Sveistrup J[50]	2014	Retro.	503(115/388)	Prostatic bed, seminal vesicles	76/2 **VS** 78/2	10 **VS** 5–7	NR	IG-IMRT	88/95p = 0.019	128/373	98.4/42	CTCAE version 4.0,ASTROPhoenix definition
Troeller A[51]*	2015	Pro.	1115(457/658)	Prostatic bed, seminal vesicles	75.6/1.8 **VS** 75.6/1.8	10	Isodose level	YES	23.2/19.9 p = 0.21	NR/NR	106.8/55.2	CTCAE version 3.0
Vora SA[52]	2007	Retro.	416(271/145)	Prostatic bed, seminal vesicles	68.4/NR **VS**75.6/NR	10–20 **VS** 6–10	NR	NO	17.6/30.3	386/30	60/48	RTOG toxicity scale, ASTROPhoenix definition
Wong WW[33]	2009	Retro.	584(270/314)	Prostatic bed, seminal vesicles	68.4/1.8–2 **VS** 75.6/NR	10–20 **VS** 6–10	NR	NO	17/36	543/41	120/120	RTOG toxicity scale, ASTROPhoenix definition
Zelefsky MJ[53]	2000	Retro.	232(61/171)	Prostatic bed, seminal vesicles	81/1.8**VS** 81/1.8	10	Isocenter	NO	34/53	194/38	39/12	RTOG toxicity scale
Zelefsky MJ[54]	2007	Retro.	1571(830/741)	NR	66-81/1.8 **VS** 81/NR	NR	Isocenter	NO	43	NR/NR	120/78	CTCAE version 3.0
Shu HK[55]	2001	Retro.	44 (26/18)	Prostatic bed, seminal vesicles	NR	7.5–10	Isodose level	NO	79.5	33/11	30.1/18.7	RTOG toxicity scale
Wortel RC[56]*	2015	RCT	475(215/260)	Prostatic bed, seminal vesicles	78/2 **VS** 78/2	10 **VS** 5–8	NR	IG-IMRT	19.5/66.9	262/213	3/3	RTOG toxicity scale
Matzinger O[57]	2009	RCT	791(652/139)	NR	70-78/2**VS** 74-78/2	NR	Isodose	NO	50	791/0	NR/NR	CTCAE version 2.0

Abbreviations: PTV = Planning target volume;retro = Retrospective study; pro = prospective study; RCT = Randomized controlled trial; ADT = Androgen deprivation therapy; NR = Not reported.

*represent studies which contain patients who underwent surgery.

Of the included studies, 14 studies compared the effects of acute toxicity of an IMRT group to that of a 3DCRT group, including acute GI toxicity (n = 12), acute GU toxicity (n = 12) and acute rectal toxicity (n = 4). Additionally, 21 studies compared the late toxicity effects of IMRTgroup to that of 3DCRT group, including late GI toxicity (n = 13), late GU toxicity (n = 12) and late rectal bleeding (n = 5). Furthermore, 6 studies compared the biochemical controlbetween IMRT group and 3DCRT group, and 3 studies compared the OS between IMRT group and 3DCRT group (Table 2).

**Table 2:** Summary of the outcomes presented in this meta-analysis.

Group	No. of studies	No. of total patients	RR (95% CI) (IMRT VS 3DCRT)	P for heterogeneity	I^2^	References
Acute GI toxicity (grade 2–4)	12	4142	0.59 (0.44, 0.78)	0.000	84.0%	[33,36–41,43,52,56–58]
Acute GU toxicity (grade 2–4)	14	4603	1.08 (1.00, 1.17)	0.026	47.2%	[33,36–41,43,45,52,53,56–58]
Acute rectal toxicity (grade 2–4)	4	2188	1.03 (0.45, 2.36)	0.005	76.8%	[45,47,53,54]
Late GI toxicity (grade 2–4)						
1 year	4	1634	0.38 (0.15, 0.97)	0.002	80.2%	[37,41,46,48]
3 years	7	2243	0.70 (0.44, 1.13)	0.004	71.3%	[37,38,40,41,43,46,48]
5–10 years	8	4900	0.55 (0.31, 0.98)	0.000	93.9%	[33,37,41,44,48,51,52,54]
Total	13	6519	0.54 (0.38, 0.78)	0.000	90.4%	[33,37,38,40,41,43,44,46,48,50–52,54]
Late GU toxicity (grade 2–4)						
1 year	3	1341	0.83 (0.64, 1.06)	0.415	0.0%	[37,41,50]
3 years	5	1815	1.00 (0.79, 1.28)	0.905	0.0%	[37,40,41,43,53]
5–10 years	8	4128	1.03 (0.69, 1.51)	0.000	83.7%	[33,37,41,44,46,48,52,54]
Total	12	5608	1.03 (0.82, 1.30)	0.000	72.3%	[33,37,40,41,43,44,46,48,50,52–54]
Late rectal bleeding (grade 2–4)	5	1972	0.48 (0.27, 0.85)	0.05	58%	[42,45,47,49,53]
Biochemical control	6	2416	1.17 (1.08, 1.27)	0.010	67.0%	[33,37,41,44,45,50,52]
OS	3	924	1.07 (0.96, 1.19)	0.009	79.0%	[37,41,44]

### Acute GI toxicity

**Fig 2:**
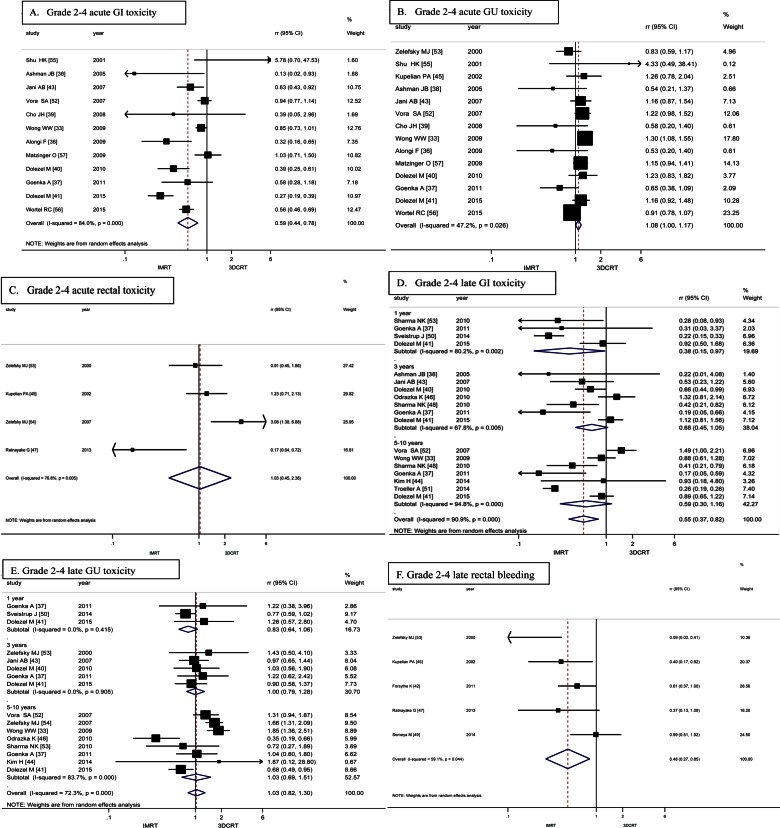
Forrest plots of RRs for IMRT versus 3DCRT about the grade 2–4 acute toxicity and late toxicity. (A) acute GI toxicity, (B) acute GU toxicity and (C) acute rectal toxicity, (D) late GI toxicity, (E) late GU toxicity and (F) late rectal bleeding.

Acute GI toxicity was investigated in 12 studies with 4142 patients [33,36–41,43,52,56–58]. Pooled RR indicated that IMRT significantly decreased grade 2–4 acute GI toxicity compared with 3DCRT [RR = 0.59, 95% CI (0.44, 0.78)] (Fig 2A). Due to obvious heterogeneity, random effect model was employed.

### Acute GU toxicity

A total of 14 studies with 4603 patients assessed the acute GU toxicity [33,36–41,43,45,52,53,56–58]. Pooled RR indicated that the incidence of grade 2–4 acute GU toxicity was only 1.08 -fold higher in IMRT than that in 3DCRT, which showed modest effect [RR = 1.08, 95% CI (1.00, 1.17)] (Fig 2B). No obvious heterogeneity was found, thus fixed effect model was performed.

### Acute rectal toxicity

Data regarding acute rectal toxicity were available in 4 studies with 2188 patients [45,47,53,54]. In those four studies, there was no significant difference between IMRT and 3DCRT in grade 2–4 acute rectal toxicity [RR = 1.03, 95% CI (0.45, 2.36)] (Fig 2C). With obvious heterogeneity observed, the random effect model was employed.

### Late GI toxicity

Late GI toxicity was discussed in 13 studies with 6519 patients [33,37,38,40,41,43,44,46,48,50–52,54]. A significant overall benefit of grade 2–4 late GI toxicity in favor of IMRT was found for all studies with a RR of 0.54 [95% CI (0.38, 0.78)] (Fig 2D). The subgroup analysis demonstrated significant differences in grade 2–4 late GI toxicity between IMRT and 3DCRT at 1 year [RR = 0.38, 95% CI (0.15, 0.97)] and 5–10 years [RR = 0.55, 95%CI (0.31, 0.98)], with a non-significant difference at 3 years [RR = 0.70, 95%CI (0.44, 1.13)]. For the obvious heterogeneity, the random effect model was performed.

### Late GU toxicity

A total of 12 studies with 5608 patients were included in meta-analysis to evaluate grade 2–4 late GU toxicity [33,37,40,41,43,44,46,48,50,52–54]. Pooled RR indicated that IMRT was with comparable grade 2–4 late GU toxicity with 3DCRT [RR = 1.03, 95% CI (0.82, 1.30)] (Fig 2E). The subgroup analysis also showed no significant difference between two treatments at 1 year [RR = 0.83, 95% CI (0.64, 1.06)], 3 years [RR = 1.00, 95% CI (0.79, 1.28)] and 5–10 years [RR = 1.03, 95% CI (0.69, 1.51)]. Due to the significant heterogeneity, random effect model was used for this analysis.

### Late rectal bleeding

Data regarding late rectal bleeding were available in 5 studies with 1972 patients [42,45,47,49,53]. The results clearly favor IMRT over 3DCRT in grade 2–4 late rectal bleeding [RR = 0.48, 95% CI (0.27, 0.85)] (Fig 2F). With obvious heterogeneity found, the random effect model was employed.

### Biochemical control

**Fig 3:**
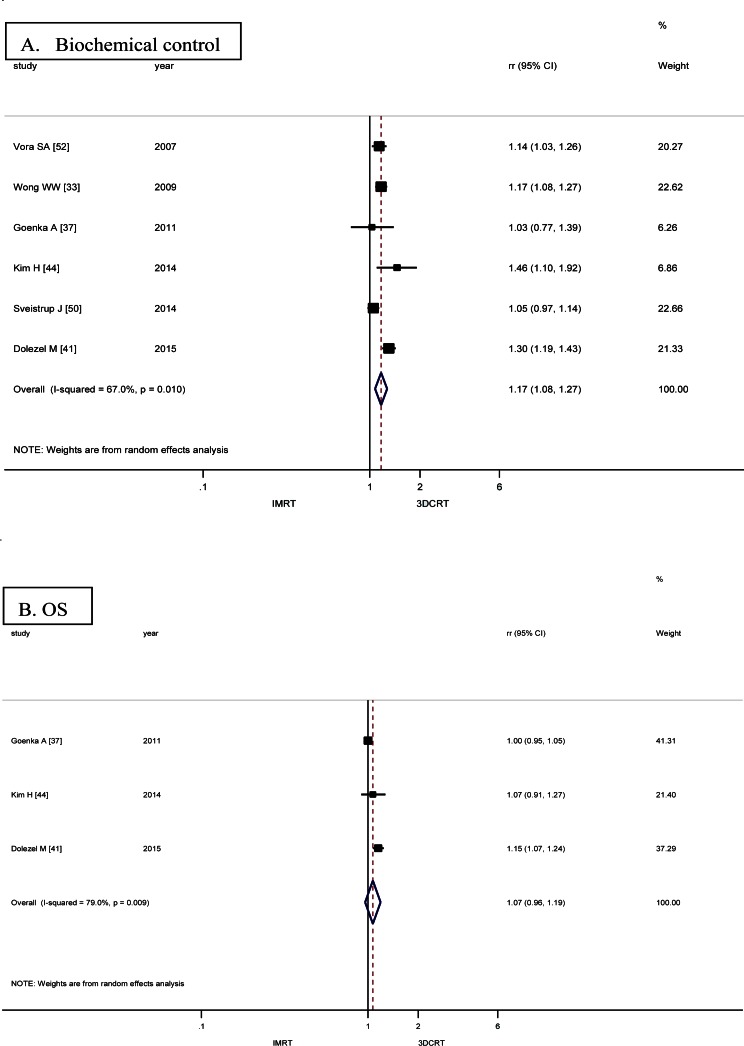
Forrest plots of RRs for IMRT versus 3DCRT about the survival outcomes. (A) Biochemical control, (B) OS.

Biochemical control was reported in 6 studies with 2416 patients [33,37,41,44,45,50,52]. There was a significant difference in biochemical control favoring IMRT [RR = 1.17, 95% CI (1.08, 1.27)] (Fig 3A). IMRT showed modest increase in biochemical control in comparison with 3DCRT. Random effect model was employed because of the significant heterogeneity.

### Overall survival

Data regarding overall survival were available in three studies with 924 patients [37,41,44]. A non-significant increase in overall survival favoring IMRT was found [RR = 1.07, 95%CI (0.96, 1.19)] (Fig 3B). Random effect model was performed for the obvious heterogeneity.

### Publication bias

**Fig 4:**
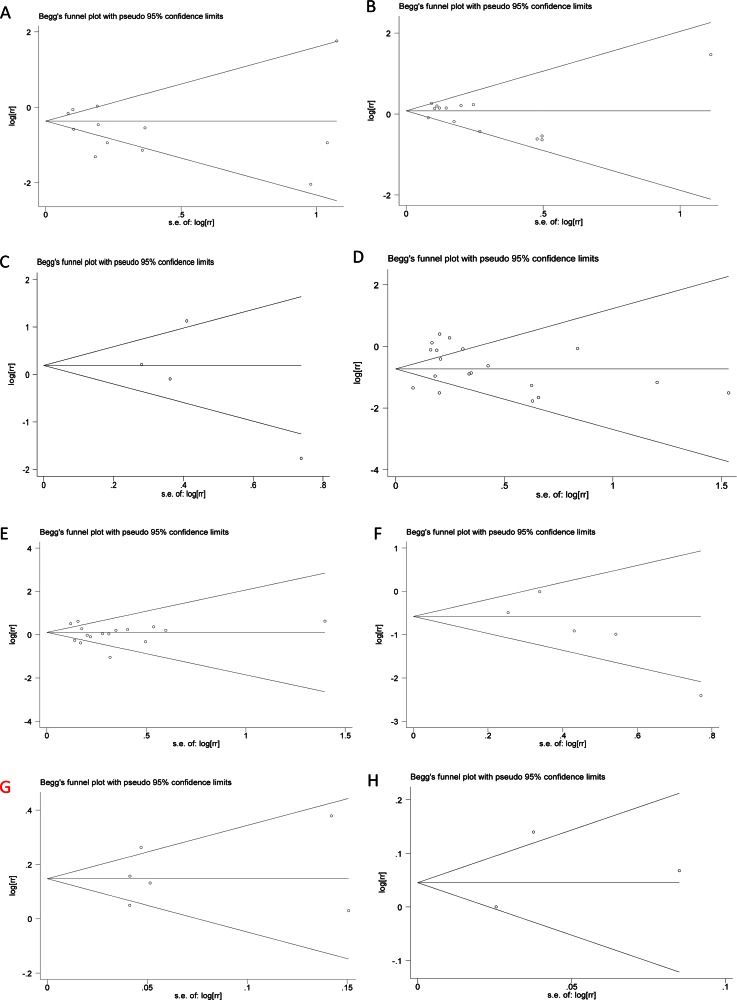
Funnel graph for assessing the potential publication bias in the studies comparing IMRT and 3DCRT in patients diagnosed with prostate cancer. (A) acute GI toxicity, (B) acute GU toxicity, (C) acute rectal toxicity, (D) late GI toxicity, (E) late GU toxicity, (F) late rectal bleeding, (G)Biochemical control, (H) OS.

Both Begg’s funnel plot and Egger’s test were employed to assess the publication bias in all studies evaluating acute GI toxicity, acute GU toxicity, acute rectal toxicity, late GI toxicity, late GU toxicity, late rectal bleeding, biochemical control, and OS, respectively (Fig 4). The Begg’s funnel plot did not indicate any evidence of statistically significant asymmetry in the meta-analysis of acute GI toxicity (p = 0.784), acute GU toxicity (p = 0.661), acute rectal toxicity (p = 0.497), late GI toxicity (p = 0.248), late GU toxicity (p = 0.787), late rectal bleeding (p = 0.142), biochemical control(p = 0.851) and OS (p = 0.602). There was also no evidence of publication bias in Egger’s test of acute GI toxicity (p = 0.271), acute GU toxicity (p = 0.345), acute rectal toxicity (p = 0.485), late GI toxicity (p = 0.335), late GU toxicity (p = 0.451), late rectal bleeding (p = 0.118), biochemical control(p = 0.682) and OS (p = 0.692).

## Discussion

In this meta-analysis, we enrolled 23 eligible studies comparing the clinical outcomes between IMRT and 3DCRT in patients diagnosed with prostate cancer. The present study showed that IMRT was associated with decreased 2–4 grade acute GI toxicity, late GI toxicity, and late rectal bleeding compared with 3DCRT. However, IMRT significantly increased grade 2–4 acute GU toxicity with similar grade 2–4 late GU toxicity. Moreover, no significant differences were discovered in grade 2–4 acute rectal toxicity and overall survival. Nevertheless, IMRT showed improved biochemical control than 3DCRT,suggesting better PSA relapse-free survival in IMRT. These results imply that IMRT might be superior to 3DCRT with less toxicity and better PSA relapse-free survival in patients diagnosed with prostate cancer. However, more high quality studies will be needed to further identify this result.

Compared with 3DCRT, IMRT can deliver radiation with the capability of intensely conforming to cancerous site, which means IMRT can deliver higher dose to the target volume with less damage of normal tissues and with the creation of steep dose gradients and concave dose distribution [59,60]. On the one hand, dose-escalated RT has been demonstrated to generate better biochemical control when compared with lower doses by some randomized trials [8,61]. On the other hand, higher doses were associated with increased RT related side effects. Therefore, IMRT is generally believed to minimize treatment related toxicity and relatively improve survival. Besides, IMRT can also be performed to increase the homogeneity of dose distribution [59].

IMRT also has some drawbacks. Compared with 3DCRT, IMRT leads to larger volumes of healthy tissues exposed to low doses of radiation, which may increase the risk of second malignancies. However, more solid data are needed to clarify the clinical relevance [27,62]. Furthermore, IMRT is a kind of complex RT technique, which needs longer delivery time and has higher requirements for the physicists [63]. IMRT is estimated to cost about £1100 more than 3DCRT, which mainly comes from additional radiographer, medical and physics staff time [64]. Nevertheless, there is still a need to understand the cost-effectiveness of IMRT, which may produce more quality-adjusted life-years (QALYs) with lower total costs [64]. Hence, it is important to assess the benefits and risks of IMRT.

In the published trials of RT, GI and GU toxicities are the most frequently studied and may deeply influence quality of life in patients who are diagnosed with prostate cancer [65–69]. Rectal bleeding is a type of late GI toxicity, but it sometimes is reported as a sole end point due to its objectivity [70–73]. Therefore, in this meta-analysis, we assessed not only the PSA relapse free survival and overall survival, but also the GI and GU toxicity and late rectal bleeding between 3DCRT and IMRT. However, randomized controlled trials that compare the clinical efficacy of IMRT with 3DCRT are still lacking.

Although meta-analysis is considered the gold standard by some authors, some potential bias cannot be completely eliminated. Begg’s funnel and Egger’s test were employed in this meta-analysis, and no statistically significant publication bias was discovered. However, several aspects which may produce potential biases in this meta-analysis should be discussed. First, only the literatures published in English were included because of the inaccessibility of other languages for reviewers. So the literature published in other languages, such as German, French and Spanish, was excluded in this meta-analysis [74–76]. This selection may cause further approval of the positive results, because positive results usually are published in English, while negative results tend to be published in native languages. This is called “file-drawer problem”. Second, some studies were excluded due to the inaccessibility of extractingestimated RR value. One example of this is a study that compares the toxicity between 3DCRT and IMRT in the treatment of localized prostate cancer. In this study, they found a significant difference in late GU morbidity between 3DCRT and IMRT (p = 0.025).However, no data was available about late GU toxicity for meta-analysis from this study [58].

Third, the obvious heterogeneity between studies may be derived from different characteristics of study design, including different sample size, tumor stage, combined therapy, previous treatments, follow-up time, dose of the radiation therapy, etc. For example, two included studies reported that all of their patients had a prostatectomy, while only 15.9% to 54.9% of the patients in the remaining studies had a prostatectomy before radiotherapy [37]. Besides, the doses of the radiation varied among studies. Most of the studies used prescribed doses of 70 to 78 Gy, while one study performed a median dose up to 85.3 Gy, which may produce a different effect on the morbidity of GU or GI toxicity [58].

One limitation of this study is that we ignored the effect of combination treatments, and were not able to stratify patients according to whether they received surgery. In those studies which contain patients who underwent surgery, only one study analyzed the relationship between surgery and the incidence of late GU toxicity[40,41,45,46,51,56]. In this study, the actuarial 5-year risk of late GU toxicity was significantly higher in patients with a history of prostatectomy than in those without surgery [HR = 2.35 (95%CI 1.17–4.71)] [46].The other studies only compared the clinical outcomes of 3DCRT with IMRT without analyzing the influence of surgery on different RT technologies. Therefore, based on the insufficient data, we can’t analyze the effect of surgery, on survival or toxicity. As for hormone therapy, only 3 studies discussed the influence of hormone therapy on late toxicity [46,47,50]. One study analyzed the influence of hormone therapy on late rectal toxicity, and concluded that hormone therapy had no significant influence on the risk of late rectal toxicity [HR = 2.59, 95% CI (1.00, 6.70), p = 0.10] [47]. Data regarding late GU toxicity was available in the other two studies [46,50]. Subgroup analysis showed no significant influence of hormone therapy on incidence of late GI and late GU toxicity ([HR = 0.47, 95% CI (0.16, 1.39)], [HR = 0.65, 95% CI (0.42, 1.01)], respectively) (S1 Fig). Therefore, we concluded that hormone therapy might have no influence on the occurrence of late GI or GU toxicity in the treatment of 3DCRT and IMRT. Another limitation is that we did not stratify the patients according to recurrence risk. Only two studies separated their patients into low, intermediate and high risk groups, which was not enough for us to perform a subgroups analysis with such small numbers of studies[41,44]. More researches investigating the associations of risk group and radiotherapy are needed.

In conclusion, IMRT significantly decreases the occurrence of 2–4 grade acute GI toxicity, late GI toxicity, late rectal bleeding, and achieves better PSA relapse free survival in comparison with 3DCRT. IMRT and 3DCRT remain the same in regard of acute rectal toxicity, late GU toxicity and overall survival, while IMRT increases the morbidity of acute GU toxicity. In general, based on the above results, IMRT should be considered as a better choice for the treatment of prostate cancer. More randomized controlled trials are needed to determine the subset of patients diagnosed with prostate cancer.

## Supporting Information

S1 PRISMA Checklist (DOC)

S1 Fig Forrest plots of HR for IMRT versus 3DCRT about the hormone therapy. (A) Late GI toxicity, (B) Late GU toxicity.
(TIF)
