# Supplementary figures and images for: The Effectiveness of Intensity Modulated Radiation Therapy versus Three-Dimensional Radiation Therapy in Prostate Cancer: A Meta-Analysis of the Literatures
Source: PLoS One. 2016 May 12;11(5):e0154499. doi: 10.1371/journal.pone.0154499 (PMC4865138; doi:10.1371/journal.pone.0154499)

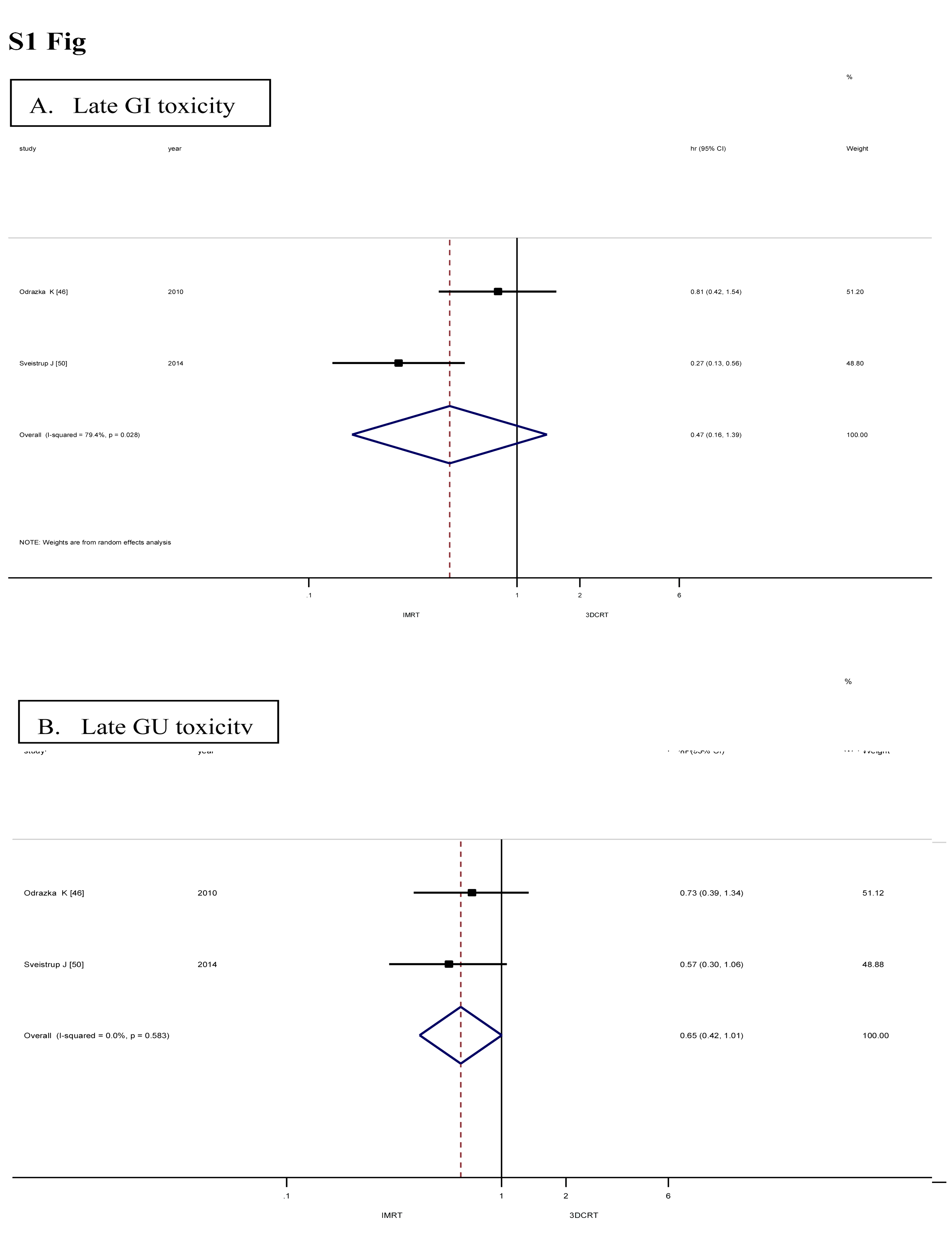

Supplement: S1 Fig — (A) Late GI toxicity, (B) Late GU toxicity. (TIF) [file pone.0154499.s002.tif]
